# Cooperative UAV Scheme for Enhancing Video Transmission and Global Network Energy Efficiency

**DOI:** 10.3390/s18124155

**Published:** 2018-11-27

**Authors:** Pedro Cumino, Wellington Lobato Junior, Thais Tavares, Hugo Santos, Denis Rosário, Eduardo Cerqueira, Leandro A. Villas, Mario Gerla

**Affiliations:** 1 Computer Science Faculty, Federal University of Pará (UFPA), Belém 66075-110, Brazil; wellingtonl@ufpa.br (W.L.J.); thaistavares@ufpa.br (T.T.); hugosantos@ufpa.br (H.S.); denis@ufpa.br (D.R.); cerqueira@ufpa.br (E.C.); 2 Institute of Computing, University of Campinas (UNICAMP), Campinas 13083-970, Brazil; leandro@ic.unicamp.br; 3 Computer Science Department, University of California Los Angeles (UCLA), Los Angeles, CA 90095-1596, USA; gerla@cs.ucla.edu

**Keywords:** energy-aware, energy-efficiency, UAV replacement, UAV coordination

## Abstract

Collaboration between multiple Unmanned Aerial Vehicles (UAVs) to set up a Flying Ad Hoc Network (FANET) is a growing trend since future applications claim for more autonomous and rapid deployable systems. The user experience on watching videos transmitted over FANETs should always be satisfactory even under influence of topology changes caused by the energy consumption of UAVs. In addition, the FANET must keep the UAVs cooperating as much as possible during a mission. However, one of the main challenges in FANET is how to mitigate the impact of limited energy resources of UAVs on the FANET operation in order to monitor the environment for a long period of time. In this sense, UAV replacement is required in order to avoid the premature death of nodes, network disconnections, route failures, void areas, and low-quality video transmissions. In addition, decision-making must take into account energy consumption associated with UAV movements, since they are generally quite energy-intensive. This article proposes a cooperative UAV scheme for enhancing video transmission and global energy efficiency called VOEI. The main goal of VOEI is to maintain the video with QoE support while supporting the nodes with a good connectivity quality level and flying for a long period of time. Based on an Software Defined Network (SDN) paradigm, the VOEI assumes the existence of a centrailized controller node to compute reliable and energy-efficiency routes, as well as detects the appropriate moment for UAV replacement by considering global FANET context information to provide energy-efficiency operations. Based on simulation results, we conclude that VOEI can effectively mitigate the energy challenges of FANET, since it provides energy-efficiency operations, avoiding network death, route failure, and void area, as well as network partitioning compared to state-of-the-art algorithm. In addition, VOEI delivers videos with suitable Quality of Experience (QoE) to end-users at any time, which is not achieved by the state-of-the-art algorithm.

## 1. Introduction

Flying ad hoc networks (FANETs) [1] are composed of a group of Unmanned Aerial Vehicles (UAVs), which are autonomous aircrafts without the need of a pilot to be on board during the flight [2]. Compared to a single UAV system, a FANET is more robust and can cover a larger area of interest [3,4]. In this context, FANETs have been employed in many kinds of new smart city scenarios, such as disaster recovery, environmental monitoring, cooperative safety and security, and others. In these scenarios, multimedia content plays an important role in helping ground rescue teams to take appropriate decisions based on detailed and rich visual information [5].

Video dissemination over FANET for such applications requires Quality of Experience (QoE) support to deliver the content with a minimal quality level based on the user perspective [6]. This is because users expect to receive live videos without ghosting, blocking, pixelization, freeze frame in order to enable the rescue team in the control center to take action to explore a hazardous area based on rich video information [7]. In this way, there is a demand for video with low frame loss rate, tolerable end-to-end delay, and low jitter to provide QoE support [8].

The UAVs will be the humans’ eyes in the sky, and thus they must be flying and monitoring events for a long period of time [9]. In this sense, energy efficiency is a key factor in FANETs in order to keep the UAVs cooperating as much as possible during a mission [10]. It is also important to maintain the delivery of videos with a better QoE while avoiding interruption periods. In general, UAVs are equipped with batteries with limited capacity, which spends energy on computing, flying, and transmitting. In many cases, it is not recommended to use solar panel or different power sources in UAVs because it can increase the payload. In order to avoid UAV loss due to the energy issue, UAVs must return to the launch point to replace or recharge their batteries. This is because UAV loss reduces the number of UAVs in the scenario, and causes route failure and void area as well as network partitioning.

In this sense, effective energy-aware deployment mechanisms are needed for timely onboard energy replenishment without interruption of the video services [2]. Specifically, UAV must leave the monitoring area for battery revamping, during which the service gap is temporarily filled by neighboring UAVs that adjust their positions without the interruption of video services. In addition, the energy-efficient operation must be taken into account through smart energy management, that is, accomplishing missions with minimum energy consumption [2]. For instance, decision-making must take into account the energy consumption associated with UAV movements since they are generally quite energy-intensive.

An intelligent coordination protocol must control the behavior of multiple UAVs, maximizing the benefits of a FANET [11]. This UAV controlling protocol manages both the UAV communication (i.e., routing protocols), as well as the UAV movements. In this context, Software-Defined Networking (SDN) [12,13] is a promising approach for FANET controlling task, since it introduces complete network programmability by separating the control plane and data plane. For instance, the UAV programmability means to perform UAV replacement, to improve the application performance, to determine the data routing paths due to performance or energy reasons. SDN also considers a centralized SDN controller responsible for all control functions, such as routing, UAVs replacement, collision avoidance, and other services. In this sense, the controller node is aware of the global network information to optimize the network operations.

This article proposes a cooperative UAV scheme for enhancing video transmission and global energy efficiency, called VOEI. VOEI performs the decision-making of a video-aware FANET by taking into account energy issues to provide energy-efficiency operations, avoiding the premature death of nodes, network disconnections, route failures, void areas, and low-quality video transmissions. VOEI supports the concept of SDN into FANET to separate the control and data plane, as well as to provide network flexibility and programmability by controlling UAVs’ operation parameters on-the-fly. The main benefits of VOEI is due to the SDN controller considers global FANET context information to establish reliable and energy-efficiency routes, and to prevent UAV loss caused by energy issue and video delivery failure. In this sense, the controller must compute reliable and energy-efficiency routes, as well as trigger the UAV replacement process to keep the FANET connectivity level (dynamic paths) with a suitable quality level. Simulation results demonstrate the efficiency of the VOEI in disseminating videos with high energy-efficiency, while achieving QoE support in a video-aware FANET scenario. The main contributions of VOEI are twofold: (i) the use of a SDN controller to control all operations in a cooperative FANET system, while improving the selection of reliable, suitable and energy-efficiency routes. Thus, VOEI aims to increase the video QoE and the network lifetime; (ii) assessment of the impact of different UAV speeds on the energy level, as well as QoE.

The remainder of the article is organized as follows. Section 2 outlines existing works for video dissemination over FANET, and their main drawbacks to support energy-efficiency and QoE. Section 3 presents the VOEI mechanism by describing its components and operations. Section 4 describes the simulation settings for the performance evaluation in a video dissemination scenario and also discusses the results. Finally, the concluding remarks are presented in Section 5.

## 2. Related Work

Surveillance and monitoring is a kind of service where camera-equipped UAVs are increasingly present. In the related environment, the high QoE of video transmitted is crucial to take a correct and precise decision, mainly in an emergency situation. The work presented by Arnaldo Filho et al. [14] shows a mechanism called of mobiFANET, which assists relay node replacement in order to guarantee a reasonable video distribution that consequently increases the QoE. MobiFANET takes into account the UAV location and mobility trajectory to establish the ideal relay node location, keeping the connectivity between neighbor UAVs of a given path, mitigating the effects of UAV mobility, avoiding communication flaws, delays, and void area. MobiFANET also mitigates the effects of UAV mobility, avoiding communication failures, delays, and empty areas. However, the limited view of the UAVs resources does not allow rearrange the FANET to provide further UAVs energy replenishment regarding maintaining QoE.

Sánchez-García et al. [15] highlighted the main research topics about exiting UAV and aquatic vehicles. Regarding communication technology, the main advantage of IEEE 802.15.4 is energy saving compared to IEEE 802.11. The first technology takes the advantage from the lower transmission rate to avoid extra energy loss in communication. However, the adoption of IEEE 802.11 is more feasible for video transmission despite the energy consumption. Another advantage is the longer distance that can be reached in a environment that the nodes can be placed far from each other. Moreover, it also demands fewer requirements when compared to satellite communications. In addition, Shinkuma [16] proposed a Wireless Mesh Networks design composed by UAVs, assuming batteries and Access Points are replaceable and separable from UAVs and both are transported and placed in appropriate positions by the mechanical automation of UAVs. The UAV nodes are equipped with IEEE 802.11g due to its simplicity to determine the channel model and transmission rate. In addition, each node works as AP and is positioned in order to enhance the network throughput.

Guillen-Perez et al. [17] presented an overview of the main characteristics of FANET. The work presents the state-of-the-art about mobility, positioning and propagation models adopted. However, the focus is on the limitations of the propagation model, which has a great impact on the communication among UAVs and consequently on network lifetime. A case study is also presented and showed the impact of radio propagation on UAV to UAV, and on UAV to Ground communication, showing that the chassis decreases 10 dB the power of radio in UAV to Ground links.

Zhao et al. [11] introduced the Software-Defined UAVNet architecture (SD-UAVNet), which implements the concept of SDN into UAVNets to separate the control and data plane, as well as to provide network programmability by controlling UAVs operation parameters. The SD-UAVNet controller considers the global UAV context information to prevent UAV collisions, optimize the UAVs’ movements, and establish a routing path, which determines the relay node deployment to provide video transmission with QoE support. However, SD-UAVNet does not consider any mechanism to provide energy-efficiency operations, such as UAV replacement.

Reina et al. [18] proposed a UAV deployment scheme based on evolutionary algorithms. The idea is to provide Internet connectivity to users in a rural disaster scenario. The approach is divided into two phases, namely initial and adaptation phases. In the initial phase, the UAV network is deployed by taking into account only partial information. On the other hand, in the adaptation phase, the UAVs move according to a searching algorithm in order to stay in a better coverage area to attend the users. The decision-making in the initial phase runs in a centralized fashion, once it requires a high computational power to run a Genetic Algorithm. It aims to find a suitable position for the UAVs with limited information. A distributed decision making is also adopted. In the second phase, the UAVs exchange information among them in order to locally calculate a better coverage position. A Hill Climbing algorithm is used, which requires less computational power, making it feasible to run on UAVS. However, it is not clear of what type of data will be retransmitted from the user by the UAVs. An excessive amount of transmitted information among the UAVs in the second phase might decrease the network lifetime. Another issue is that, in the real world, the proposal might not be feasible once it does not consider the lifetime of the battery.

Acevedo et al. [19] introduced a distributed solution for long duration area surveillance missions, which takes into account limitations in the communication between the nodes in a set of UAVs. Surveillance application must operate independently of the necessity of group presence and maintenance. In addition, in monitoring missions, the presence and maintenance of the group are necessary, without impacting the functionality of the surveillance application. Each node considers its own information and its neighbors, so it is more difficult to have a global view of the energy consumption of each device, and the choice of nodes that can replace the battery. However, the proposed approach does not maintain the UAV coordination regarding energy efficiency influence.

The need for refueling or recharging in intrusion detection applications has been extensively analyzed. In this case, Burdakov et al. [20] proposed a mechanism for the problem of scheduling the replacements of the UAVs, seeking to optimize the operation of the application. For this, the authors propose a scenario where there is a set of battery-operated UAVs, each one performing routes to maximize the probability of detecting an attacker. Once a UAV node leaves the detection area, another node is allocated with the battery fully charged. The work focuses on the scheduling of the recharges and does not consider the communication between the UAVs, to make the scheduling decisions of the substitution strategy. Moreover, the solution developed is an algorithm that has information of all the active and reloading nodes, which seems like a nearly unfeasible approach.

A single UAV equipped with a camera has been used to support structural inspection, such as introduced by Erdelj et al. [9]. As a UAV has a short time of autonomy and structural inspection usually requires a long period of time to accomplish a reasonable result, the solution attempts maintain the service by replacing the UAV node in a transparent manner. The basic infrastructure presented is composed of a Ground Control Station (GCS) and a UAV. A machine state approach protocol that runs in the network is developed and is in charge of sending a full recharged UAV to the place where the drained UAV is present, exchanges the service-sensitive information and then sends the drained UAV to the GCS in order to recharge it. However, the approach does not consider any aspects of cooperation to grant network stability and large area video service coverage.

Based on our analysis of the state-of-the-art, we conclude that it is essential to provide energy-efficiency operations in video dissemination over FANETs, avoiding the premature death of66nodes, network disconnections, route failures, void areas, and low-quality video transmissions. In this way, UAV replacement must be provided in order to avoid the premature death of nodes, network disconnections, route failures, void areas, and low-quality video transmissions. However, so far, not all of these key features have been provided in a unified cooperative UAV scheme for enhancing the video transmission.

## 3. Cooperative UAV Scheme for Enhancing Video Transmission and Global Network Energy Efficiency (VOEI)

VOEI aims to improve the cooperation of UAVs for enhancing video transmission and global network energy efficiency in global FANETs. The main goal is to maintain the video with QoE support while supporting the nodes with a good connectivity quality level and flying for a long period of time. Based on an SDN architecture, the VOEI assumes the existence of a controller node to compute reliable and energy-efficiency routes, as well as to coordinate the movement and UAV replacement/recharge. In the following, we describe the considered network and system model, and also the VOEI operation.

### 3.1. Network and System Model

**Figure 1:**
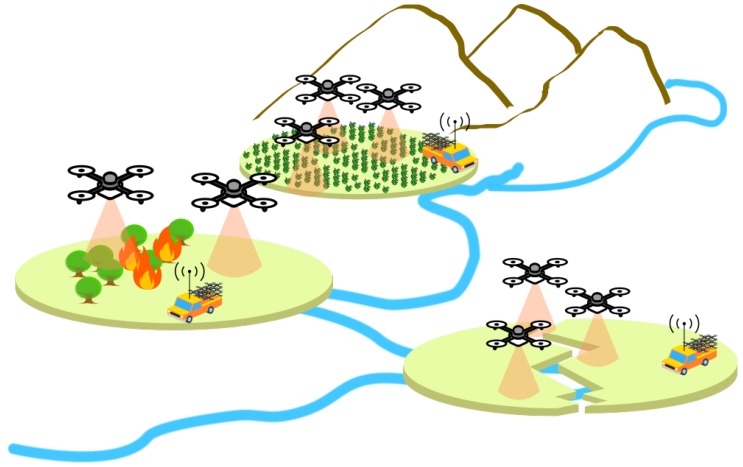
Video dissemination over FANET for disaster recovery scenario.

FANETs will be an important resource in smart cities to monitor events by transmitting video flow from the UAVs to the ground team or service, for instance, monitoring an earthquake or hurricane, as depicted in Figure 1. In this way, video captured from the event area enable humans (or even a service) in the control center to take appropriate action based on rich visual information in order to explore a hazardous area, where rescuers are unable to reach easily and quickly.

**Figure 2:**
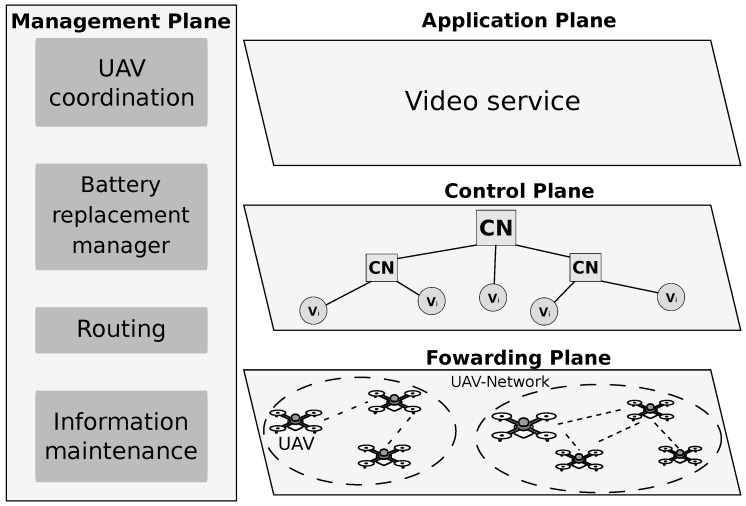
SDN architecture.

We consider the concept of SDN applied into FANET, such as introduced by Zhao et al. [11]. In this sense, the SDN paradigm provides flexibility to network management by separating the network infrastructure into distinct planes, where each plane can be programmed to meet particular application requirements [21]. Following the SDN paradigm, we consider four planes (application, control, forwarding, and management planes), and a centralized controller node as shown in Figure 2.

The application plane is on the top of the proposed architecture to support multimedia applications in FANET scenario. On the other hand, the forwarding plane is located at the bottom of the SDN architecture and it is composed of (re)configurable nodes connected to a centralized controller. The control plane is located at the middle of the network architecture, which provides functions that determine the behavior and performance of the network. The Controller Node (CN)⊂V is responsible for all control functions for managing the mission-specific decisions, such as UAVs replacement, routing, and others. The controller CN could be a ground station or a regular UAV node. For instance, a fixed-wing UAV has more energy and larger radio range, which can periodically collect UAV information and send configuration commands. In addition, for large-scale environments, it might be necessary to deploy multiple local Controller nodes that are responsible only to forward the control message to/from the main CN. This enables the proposed scheme, to transmit control messages with lower transmission power, which leads to reduced interference and energy consumption, as well as better control message reliability.

In this multimedia FANET scenario, we consider *n* UAVs (nodes) deployed in the monitored area, and each UAV has an individual identity (*i*∈[1,n]). Those UAVs are represented in a dynamic graph G(V,E), where the vertices $V=\{v_{1},\cdots ,v_{n}\}$ mean a finite set of UAVs, and edges $E=\{e_{1},\cdots ,e_{n}\}$ build a finite set of asymmetric wireless links between 1-hop UAV $\left(v_{i}\right)$ neighbors. We denote $N(v_{i})\subset V$ as a subset of all 1-hop neighbors within the radio range $\left(R_{max}\right)$ of a given UAV $v_{i}$. Each UAV $v_{i}$ has a queue (*Q*) with a maximum queue capacity ($Q_{max}$). The queue policy schedules the packet transmission by using the First In First Out (FIFO) algorithm and drops packets using the Drop Tail algorithm.

Each UAV $v_{i}$ is equipped with a camera, an image encoder, a radio transceiver, and limited energy supply. For convenience of notation, we denote SN⊂V (Source Node) as the UAV $v_{i}$ responsible for capturing video flows and transmitting them to the Destination Node (DN)⊂V, i.e., ground station, in a multi-hop fashion via multiple relay nodes ($RN_{i}$ ⊂ *V*). We assume a FANET scenario composed of one static DN equipped with a radio transceiver, an image decoder, and unlimited energy, which is responsible for receiving the video for further processing and dissemination. Each UAV $v_{i}$ is aware of its own location $L(X_{i},Y_{i})$ by means of a positioning system, e.g., GPS or Galileo. On the other hand, the location of the DN is assumed to be known a priori by each node $v_{i}$, since it is a static node.

Each UAV $\left(v_{i}\right)$ flies with a given speed $s_{i}$ ranging between a minimum (e.g., $s_{min}$) and a maximum (e.g., $s_{max}$) speed limit towards a given trajectory ($traj_{i}$). We consider that UAVs fly following the Paparazzi mobility model [22] since it enables UAVs to adapt to any type of mission, and also groups most possible UAV movements by changing the probability of each movement type as needed [23]. Particularly, this mobility model considers five possible trajectories ($traj_{i}$): Stay-At (i.e., UAV flies in a circle), Way-point (i.e., UAV flies following a straight line to a destination position), Eight (i.e., UAV trajectory has the 8 form around two fixed positions), Scan (i.e., UAV performs a scan in an area defined by two points along the round trip trajectories); and Oval (UAV trajectory has an oval form).

Each UAV $v_{i}$ has a battery with initial energy ($E_{0}$), and it spends energy to transmit a packet ($E_{tx}$), to receive a packet ($E_{rx}$), to retrieve video frames ($e_{frame}$), and to fly with a given speed $s_{i}$ ($E_{fly}\left(d,s_{i}\right)$). Each UAV $v_{i}$ is able to measure the current energy ($E_{t}$) at any time, and thus it can compute the remaining energy ($RE_{i}$ ∈ [0, 1]) as follows:

(1) $$RE_{i}=\frac{E_{t}}{E_{0}}.$$

The energy consumed in a straight flight $E_{fly}$ to move a distance *d* at a given speed $s_{i}$ can be computed as the integral of the power $P(s_{i})$ in function of the given speed $s_{i}$ along the time [24]:

(2) $$E_{fly}\left(d,s_{i}\right)=\int_{t=0}^{t=d/s_{i}}P(s_{i})dt=P\left(s_{i}\right)\frac{d}{s_{i}}.$$

The energy required to transmit ($E_{tx}$) and to receive ($E_{rx}$) a packet depends on the transmission power ($P_{t}$). The energy consumed by the UAV $v_{i}$ to transmit a packet of length *n* bits over the wireless link with a bit rate equal to *r* bps in a time *T* is computed as [25]:

(3) $$E_{tx}=\left(P_{ct}+\frac{P_{t}}{epa}\right)T=\left(P_{ct}+\frac{P_{t}}{e_{pa}}\right)\frac{n}{r},$$

where $P_{ct}$ is the power required to run the transmitting circuit, and $e_{pa}$ is the efficiency of the power amplifier. The energy consumed by the UAV $v_{i}$ to receive a packet $E_{rx}$ is computed as [25]:

(4) $$E_{rx}=\frac{P_{cr}}{r}n,$$

where $P_{cr}$ is the power required to run the receiving circuit.

It should be highlighted that UAV movements require much more energy than for packet transmissions. In our scenario, we consider a battery replacement location $L(X_{r},Y_{r})$ that a given UAV $v_{i}$ could fly to replace/recharge the battery, and then return to the monitoring environment. Table 1 summarizes the main symbols used in this article.

### 3.2. VOEI Operation

The CN must be aware of each UAV contextual information, such as, remaining energy $RE_{i}$, location information $L(X_{i},Y_{i})$, mobility trajectory $traj_{i}$, speed $s_{i}$, etc. In this sense, the CN could define a reliable and energy-efficiency routes, as well as detect the appropriate moment for UAV replacement, i.e., battery replacement/recharge or replace a given relay node $RN_{i}$ in a route. In this sense, it triggers the UAV replacement process to keep the FANET connectivity level (dynamic paths) with a suitable quality level. Based on the collected information, the CN could detect an event at a given location. Afterwards, the CN establishes a route $R_{SN,DN}$ between source SN and destination DN nodes via multiple relay nodes $RN_{i}$ based on UAV contextual information and the event location.

#### 3.2.1. Route Establishment

The CN aims to select as SN the UAV closest to the event area in order to disseminate the video, and such node must have enough energy to capture and transmit the video. This is because we aim to retrieve the video from the event as soon as possible without running out the battery of the SN. On the other hand, the set of relay node $RN_{i}$ must be closer to the ideal relay node location in order to reach it faster without consuming a lot of energy. In both cases, if needed, the UAV must have enough energy to fly back to the control center for battery replacement.

The CN must compute the time to move $t_{move}$, which means the time needed to fly from the current location $L(X_{i},Y_{i})$ to the event location for source node SN selection or to the ideal relay node location L(X,Y) for relay node $RN_{i}$ selection. We consider a video surveillance application in a disaster recovery scenario, which requires real-time video transmissions with low packet loss ratio. Hence, UAVs that act as source or relay nodes must fly at the maximum speed $S_{max}$, since we aim to disseminate videos from the event location as soon as possible, and thus UAVs must fly with maximum speed $s_{max}$ to quickly reach the event location. In addition, we avoid selecting UAVs with time to move $t_{move}$ value higher than a defined threshold (called of $t_{max}$), reducing the number of possible SN or $RN_{i}$. Finally, we save energy by selecting UAVs with lower Time to move $t_{move}$ value, since we reduce UAV movements that consume a lot of energy. Time to move $t_{move}$ is computed as follows:

(5) $$t_{move}=\frac{dist(L\left(X_{i},Y_{i}\right),L\left(X,Y\right))}{S_{max}}.$$

The CN performs energy-aware decision by considering the minimal energy $E_{min}$ value, which means the energy required to transmit packets ($E_{tx}$), to retrieve video frames ($E_{video}$), to fly to the event or ideal location ($E_{fly}$), and to fly back to the control centre for battery replacement ($E_{replacement}$). The CN only considers a given UAV as a possible source node SN or relay node $RN_{i}$ as soon as the UAV has the current remaining energy $RE_{i}$ higher than the minimal energy $E_{min}$, which means that such UAV has enough energy to perform the defined tasks, providing energy-efficiency. It is important to mention that the energy required to become a source node SN is higher than the energy required to become a relay node $RN_{i}$ since the source node SN spends energy $E_{frame}$ to capture/encode each video frame and not only to transmit each video packet $E_{tx}$. The minimal energy $E_{min}$ for each UAV $v_{i}$ is computed as follows:

(6) Emin=iiiiiiEvideo+EtxSN+Efly+Ereplacement,if SN,iiiiiiEtxRN+Efly+Ereplacement,otherwise.

It is possible to compute the number of frames for a given video by considering video duration (denoted as $video_{duration}$) and configuration information, such as frames per second (fps). In this way, the energy required to retrieve the video frames ($E_{video}$) by the source node SN can be computed by considering the number of frames for a given video and energy required to transmit each video frame $P_{frame}$, which is computed as follows:

(7) $$E_{video}=\frac{video_{duration}}{fps}\times E_{frame}.$$

Each relay node $RN_{i}$ needs a given energy to transmit a given number of k multimedia packets, which is computed as follows:

(8) $$E_{tx}^{RN}=k\times E_{tx}.$$

Both source node SN and relay node $RN_{i}$ require a given energy $E_{fly}^{SN,RN}$ to fly from their current location $L(X_{i},Y_{i})$ to the event location or ideal relay node location L(X,Y). The required energy to fly to such location depends on the Euclidean distance ($dist_{L\left(X_{i},Y_{i}\right),L\left(X,Y\right)}$) between both location and the flying speed $s_{max}$, which is computed as follows:

(9) $$E_{fly}^{SN,RN}=E_{fly}\left(dist_{L\left(X_{i},Y_{i}\right),L\left(X,Y\right)},s_{max}\right).$$

Any UAV $v_{i}$ requires a given energy $E_{replacement}$ to fly from their current location $L(X_{i},Y_{i})$ to the battery replacement location $L(X_{r},Y_{r})$. The required energy to fly to such location depends on the Euclidean distance ($dist_{L\left(X_{i},Y_{i}\right),L\left(X_{r},Y_{r}\right)}$) between both location and the flying speed $s_{i}$, which is computed as follows:

(10) $$E_{replacement}=E_{fly}\left(dist_{L\left(X_{i},Y_{i}\right),L\left(X_{r},Y_{r}\right)},s_{i}\right).$$

In this way, the CN computes a cost function $C_{SN/RN}$ for all UAVs $v_{i}$ based on Equation (11), and thus it selects the source node SN and a set of relay node $RN_{i}$. Specifically, the CN selects the UAV $v_{i}$ with the lowest value to become the SN and $RN_{i}$, since these nodes fulfill the requirements mentioned above:

(11) CSN/RN=min=∀vi∈V=iiiiii1,if tmove>tmax,iiiiii1,if REt>Emin,iiiiiiw1×tmove+w2×Emin,otherwise.

#### 3.2.2. UAV Replacement

Energy-awareness is a key factor in FANETs in order to keep the UAVs cooperating as much as possible during a mission, as well as to maintain the delivery of videos with a better QoE while. In this way, the CN is aware of the remaining energy $RE_{i}$ of all UAVs, enabling to detect that a given UAV is running out its energy supply. In such case, the UAV must fly to the battery replacement location $L(X_{r},Y_{r})$ in order to replace the battery.

Periodically, the CN computes the required energy $E_{replacement}$ to fly from their current location $L(X_{i},Y_{i})$ to the battery replacement location $L(X_{r},Y_{r})$ for all UAVs based on Equation (10). In this way, a given UAV $v_{i}$ must fly for the battery replacement location $L(X_{r},Y_{r})$, as soon as it has the remaining energy $RE_{i}$ value closer the required energy to fly for battery replacement $E_{replacement}$. This is because, without flying to the battery replacement location, such UAV $v_{i}$ will run out of the battery resources, reducing the number of UAVs in the scenario, causing route failure and void area, as well as causing network partitioning. In addition, a given UAV $v_{i}$ flying for battery replacement cannot be selected as a relay node $RN_{i}$ or source node SN.

A given source node SN or relay node $RN_{i}$ could also require battery replacement, even computing the minimal energy $E_{min}$ to become source node SN or relay node $RN_{i}$ based on Equation (6). This is because minimal energy $E_{min}$ does not consider the energy for computing tasks, to receive packets, and also other sources of energy depletion. In this case, the CN must detect such situation and perform the source or relay node replacement. The CN computes the cost function $C_{SN/RN}$ for all UAVs $v_{i}$ based on Equation (11) in order to select a new source or relay node. It is important to mention that source or relay node with low energy only starts to fly for the battery replacement after the selected UAV arrives closer to its location, avoiding route failure during video transmission.

## 4. Evaluation

In this section, we present the simulations to demonstrate the efficiency of the VOEI in transmitting video over FANET with satisfactory QoE. Specifically, we present the methodology and metrics applied to evaluate the VOEI for video surveillance applications in a disaster recovery scenario. We evaluated the impact of different UAV speeds on the energy level, as well as QoE.

### 4.1. Simulation Description and Metrics

We implemented the VOEI protocol on the Mobile Multi-Media Wireless Sensor Network (M3WSN) OMNeT++ framework [26]. M3WSN framework implements the full network stack for FANET communications, including the physical, MAC, routing, and application layers, as well as the energy consumption and mobility, following the network and system model introduced in Section 3.1. We conducted 33 simulation runs with different randomly generated seeds, and the results show the values with a confidence interval of 95%. The simulations last for 1000 seconds (s) and run with the lognormal shadowing path loss model. We set the simulation parameters to allow wireless channel temporal variations, link asymmetry, and irregular radio ranges, as expected in a real FANET scenario.

We consider a FANET scenario composed of 35 UAVs, where 29 UAVs are flying following the PPRZM [22] over the entire flat terrain of 200 × 200 m [5] to explore and disseminate live video streaming from the environment. Such UAVs are flying with different speed limit intervals: (i) 1 to 5 m/s; (ii) 5 to 10 m/s; (iii) 10 to 15 m/s; (iv) 15 to 20 m/s. As expected in FANET multimedia applications such as safety and security, environmental monitoring, and natural disaster recovery, we have one fixed Destination Node (DN) located at (100,0) [14]. We consider one UAV located at (100,100), working as controller node CN, to periodically collect UAV information and send configuration commands. In addition, we have four UAVs responsible only for forwarding a control message for the central controller (CN). Furthermore, all UAV nodes are equipped with IEEE 802.11g [16,17]. The transmission power is set to 12 dBm, resulting in a nominal $R_{max}$ of 55 meters. Based on the simulation area and $R_{max}$, videos are received at the DN via one to four relay nodes $RN_{i}$, depending on the routing protocol routing protocol, and also the source node location. It is important to highlight that video transmission over ad hoc networks with high number of hops suffer of high packet loss ratio, while reducing the QoE [27]. Each UAV is equipped with a battery with an initial energy of 18,720 Joules.

In the simulation, UAVs rely on the Carrier Sense Multiple Access with Collision Avoidance (CSMA/CA) protocol for Medium Access Control (MAC) layer, without using Request to Send/Clear to Send (RTS/CTS) messages and retransmissions. In case of buffer overflow, UAVs consider a drop tail mechanism to drop packets. At the application layer, UAVs take into account a QoE-aware redundancy mechanism to add redundant packets only to priority frames [5]. We have conducted simulations with two setups: SD-UAVNet and VOEI protocol. Particularly, SD-UAVNet considers the SDN paradigm applied to FANET, where the controller node considers the global UAV context information to establish the route data path with relay location optimization without considering energy for decision-making [11]. On the other hand, VOEI protocol performs all decision-making in a controller by taking into account energy issues in order to provide energy-efficiency operation, such as introduced in Section 3.

We scheduled a random event at a different location, and when a given UAV detects such event, it starts to capture and disseminate a video about the event. We considered video sequences with different video features downloaded from the YUV video trace library and YouTube [28], i.e., Container, UAV${}_{1}$, and UAV${}_{2}$. We consider videos with different features since small differences in motion and complexities level can influence the obtained QoE values [29,30]. Specifically, the Container video has similar characteristics as a UAV hovering in a given area to capture the video, which means that there is a small moving region of interest on a static background. On the other hand, $UAV_{1}$ and $UAV_{2}$ videos are captured from a UAV flying in a city and in a rural environment, but $UAV_{2}$ has a higher motion level than $UAV_{1}$ caused by UAV instability during the flight. We encoded those videos with an H.264 codec at 300 kbps, 30 fps, Group of Pictures (GoP) size of 20 frames, and common intermediate format (352 × 288 pixels). The decoder applies a Frame-Copy method for error concealment to replace each lost frame with the last received one, reducing frame loss and maintaining the video quality.

We consider the number of alive nodes and the remaining energy of alive UAVs to measure the effects of energy consumption on the UAV flying and packet transmissions. In terms of video quality evaluation, QoS schemes alone are not enough to assess the quality level of multimedia applications because they fail in capturing subjective aspects of video content related to human experience [6]. QoE metrics overcome those limitations, and thus we rely on a well-known objective QoE metrics, namely Structural Similarity (SSIM). Specifically, SSIM ∈ [0,1] is based on a frame-by-frame assessment of three video components, i.e., luminance, contrast, and structural similarity. Higher SSIM values mean better video quality. We used the MSU Video Quality Measurement Tool (VQMT) to measure the SSIM values for each transmitted video.

### 4.2. Simulation Results

**Figure 3:**
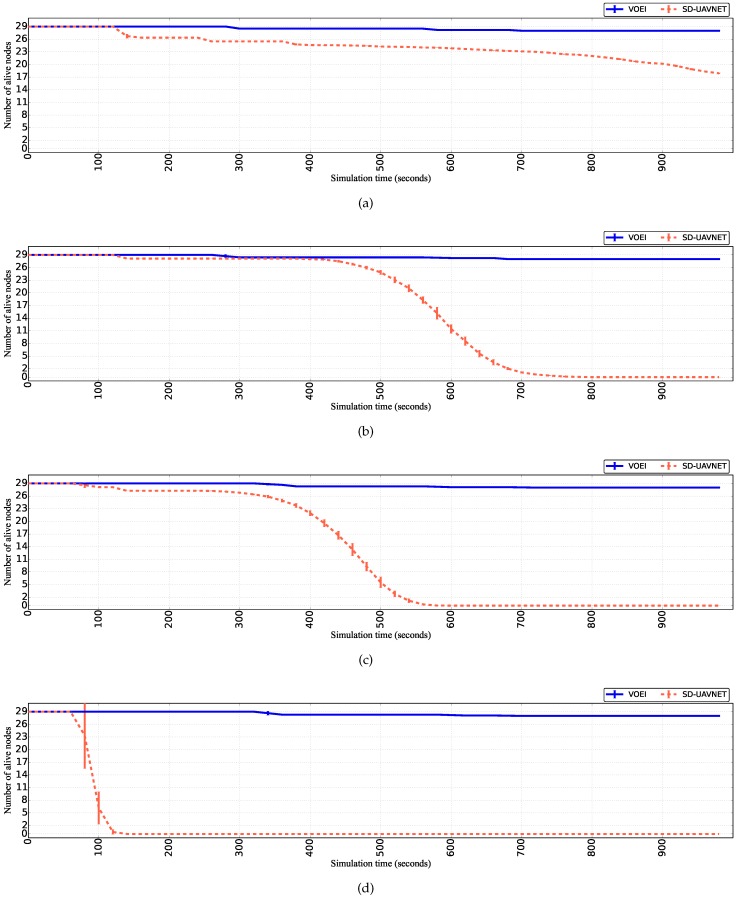
Number of live UAVs over time for UAVs flying with different speed limits. (**a**) UAVs flying with speed raging from 1 to 5 m/s; (**b**) UAVs flying with speed raging from 5 to 10 m/s; (**c**) UAVs flying with speed raging from 10 to 15 m/s; (**d**) UAVs flying with speed raging from 15 to 20 m/s.

Figure 3 shows the number of alive UAVs for both SD-UAVNet and VOEI in a scenario that UAVs are fly with different speed limits. By analyzing the results of Figure 3a, we can conclude that the first UAV to finish its energy resource occurs at the simulation time of 140 s for UAVs considering SD-UAVNet and flying with speeds ranging from 1 to 5 m/s. During this simulation time, such UAV was selected as the source node SN to capture the video and as the relay node $RN_{i}$, as well as it flew with speed closer to the maximum speed limits (i.e., 5 m/s) most of the time. Hence, such UAV consumed more energy compared to other UAVs, leading it to finish its energy resources at the simulation time of 140 s. In addition, the simulation finished with about 50% of the initial number of UAVs by considering the SD-UAVNet with UAVs flying with speeds ranging from 1 to 5 m/s. On the other hand, the simulation finished with almost of all UAVs by considering VOEI for UAVs flying with speeds ranging from 1 to 5 m/s. This is because VOEI establishes energy-efficiency routes and prevents UAV loss caused by energy issue. For instance, VOEI detects the appropriate moment for UAV replacement of UAVs with lower energy resources. Hence, VOEI provides energy-efficiency operations, avoiding network death, route failure, and void area, as well as network partitioning compared to SD-UAVNet.

As mentioned before, UAV movements require much more energy than for packet transmissions. In this way, as soon as the speed limits increase, the time that the first UAV die reduces, as well as the time that all UAVs finishes the energy resources for the scenario with SD-UAVNet. This can be seen by comparing the results of Figure 3a–d. For instance, the simulation time that the first UAV finishes its energy source reduced from 140 s for UAVs flying with lower speed (i.e., 1 to 5 m/s) to 70 s for UAVs flying with higher speed limits (i.e., 15 to 20 m/s) by considering SD-UAVNet. In addition, all UAVs finished the energy resources at the simulation time of 140 s for UAVs flying with speed ranging from 15 to 20 m/s, which does not happen for UAVs flying with lower speed limits (i.e., 1 to 5 m/s) for the scenario with SD-UAVNet. It is also important to mention that some UAVs (i.e., 1–3) finished the energy resources during the simulation with VOEI, regardless of the flying speed. This is because such UAVs must wait for the source or relay node replacement before starting the UAV replacement task, which avoids route failure during video transmission (i.e., without interruption of the video services). This is because the main goal is to disseminate video in cases of an event and not only save energy.

**Figure 4:**
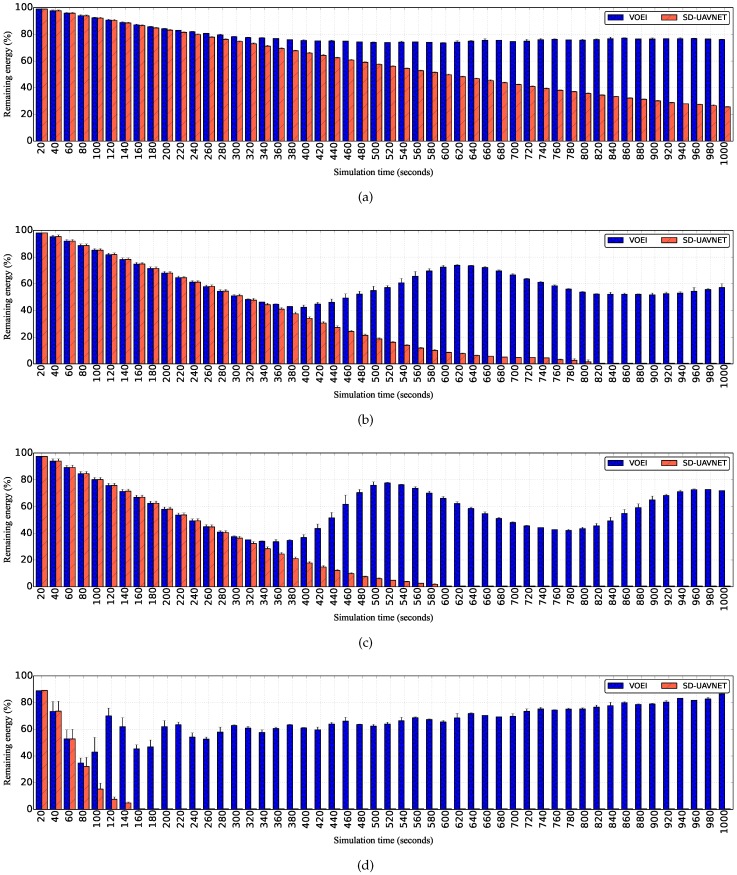
Remaining energy over time for UAVs flying with different speed limits. (**a**) UAV flying with speed raging from 1 to 5 m/s; (**b**) UAV flying with speed raging from 5 to 10 m/s; (**c**) UAV flying with speed raging from 10 to 15 m/s; (**d**) UAV flying with speed raging from 15 to 20 m/s.

Figure 4 shows the remaining energy for UAVs flying with different speed limits for both SD-UAVNet and VOEI. By analyzing the results of Figure 4, we can conclude that the remaining energy of UAVs considering SD-UAVNet linearly reduces during the simulation time, regardless of the flying speed. This is because it does not consider UAV replacement operations. On the other hand, the remaining energy of UAVs considering VOEI decreases and increases during the simulation time, since VOEI analyzes all the time if each UAV has enough energy to fly back to the control center for battery replacement. Hence, as soon as the remaining energy $RE_{i}$ of a given UAV is closer to the required energy for battery replacement, such UAV must perform the UAV replacement operations in order to prevent UAV loss caused by energy issue, which will cause video delivery failure.

**Figure 5:**
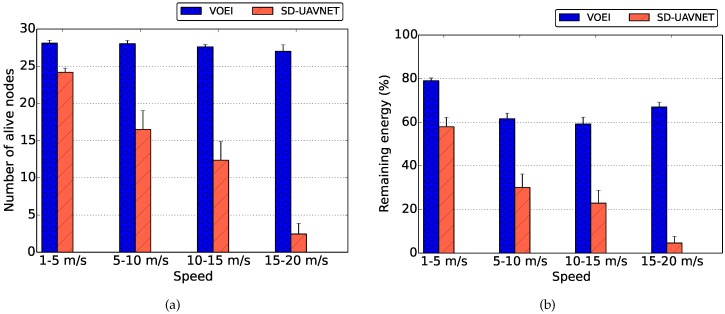
Remaining energy and number of live UAVs during the entire simulation for UAVs flying with different speed limits. (**a**) Nodes of live UAV; (**b**) Remaining Energy.

Figure 5 summarizes the average number of live UAVs and remaining energy during the entire simulation time for UAVs flying with different speed limits for both SD-UAVNet and VOEI. By analyzing the results of Figure 5a, we can conclude that VOEI provides FANET operations without reducing the number of UAVs, regardless of the flying speed. On the other hand, we can see that the number of live UAVS reduces as soon as the speed limits increases for SD-UAVNet scenario. This is explained by the results of Figure 4.

By analyzing the results of Figure 5b, we can see that the remaining energy of UAVs considering VOEI remains higher than 60% in all flying speeds. This is because CN considers global FANET context information to establish reliable and energy-efficiency routes, which prevent UAV loss caused by energy issues such as explained before. On the other hand, SD-UAVNet tends to spend more energy as soon as the speed limits increases, leading to the premature death of all UAVs as shown in Figure 5a. This is because the energy consumption is higher in high speed limits, and SD-UAVNet does not consider UAV replacement or energy-efficiency operations.

**Figure 6:**
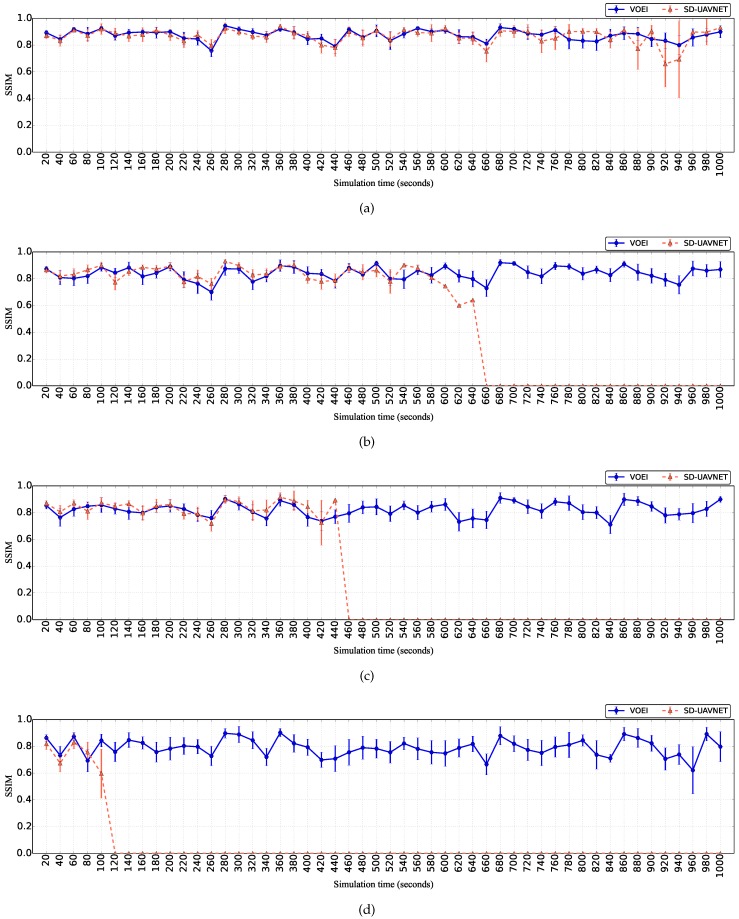
SSIM over time for UAVs flying with different speed limits. (**a**) UAV flying with speed raging from 1 to 5 m/s; (**b**) UAV flying with speed raging from 5 to 10 m/s; (**c**) UAV flying with speed raging from 10 to 15 m/s; (**d**) UAV flying with speed raging from 15 to 20 m/s.

Figure 6 shows the video quality level measured by the SSIM metric during the simulation time for videos delivered by SD-UAVNet and VOEI, while UAVs flying with different speed limits. By analyzing the results of Figure 6, we can conclude that videos delivered by SD-UAVNet and VOEI have a similar quality level. This is because both establish a route between the source and destination in a similar way. However, it is important to highlight that, in some cases, videos delivered by SD-UAVNet have higher variation in the video quality compared to videos delivered by VOEI. This is because SD-UAVNet does not consider battery replacement of UAVs with lower energy level and also relays replacement, which leads to the UAV loss caused by energy issues. Such UAV can be responsible for relaying packets for a given video, and thus it might cause route failure and packet loss. In this sense, such video will not be delivered with a good quality level due to route failure, increasing the video quality variation.

Videos delivered by VOEI have similar quality during the entire simulation, regardless of the flying speed. This is because VOEI provides the cooperation of UAVs for enhancing video transmission and global energy efficiency by introducing a battery replacement of UAVs with lower energy level and also relay replacement. It is important to highlight that VOEI is able to deliver videos with QoE support during the entire simulation, regardless of the flying speed, which is not achieved by SD-UAVNet. This is due to the controller node in VOEI being responsible for avoiding network disconnections, route failures, and void areas caused by the death of UAVs.

From our performance evaluation analysis, we conclude that VOEI improves energy-efficiency operations to deliver videos with QoE support over FANET compared to SD-UAVNet. In this sense, it can effectively mitigate the energy challenges of FANET, since it provides energy-efficiency operations, avoiding network death, route failure, and void area, as well as network partitioning compared to SD-UAVNet. In addition, VOEI delivers videos with QoE support during the entire simulation, which is not achieved by SD-UAVNet.

## 5. Conclusions

This article introduced a Video transmission with UAV replacement based on Energy Information called VOEI. It performs all decision-making by taking into account energy issues in order to provide energy-efficiency operations, avoiding network death, route failure, and void area as well as network partitioning. It computes reliable and energy-efficiency routes, as well as detects the appropriate moment for battery replenishment of UAVs, and also UAV replacement in a given route by considering global UAV context information. Simulation results demonstrate the efficiency of the VOEI in disseminating videos with high energy-efficiency, while achieving QoE support in a multimedia FANET scenario, being suitable in surveillance and disaster scenarios.

In summary, the energy-efficiency operation regarding battery refilling avoided UAV death of nearly 100% during the simulation time. The remaining energy decreased and increased over time due to UAV replacement scheme considering all speeding ranges. Our approach showed improvements in the network resilience due to the number of nodes alive with remaining energy higher than 80% also considering all speeding ranges. Moreover, the network resilience could enhance the QoE because of the continuous coverage and cooperation of the UAVs during the whole simulation time. 

## Figures and Tables

**Table 1:** List of main symbol used for VOEI description.

Symbol	Description
CN	Controller Node
$C_{SN/RN}$	Cost function for the CN to select relay and source nodes
DN	Destination node
$E_{0}$	Initial UAV energy
$E_{fly}^{SN,RN}$	Energy required to fly to an event or ideal location
$E_{fly}\left(d,s_{i}\right)$	Energy required to move a certain distance *d* with speed $s_{i}$
$E_{min}$	Energy required to trigger the battery replacement operation
$E_{replacement}$	Energy required to fly back to the control center for battery replacement
$E_{rx}$	Energy spent for receiving a packet with length *n* bits and with a bit rate of *r* bps
$E_{t}$	Current energy
$E_{tx}^{RN}$	Energy required to transmit packets for a given video
$E_{tx}$	Energy required to transmit a packet of length *n* bits and with a bit rate of *r* bps
$E_{video}$	Energy required to retrieve video frames
L(X,Y)	Ideal relay node location, where *X* and *Y* are the ideal coordinates
$L(X_{i},Y_{i})$	Current UAV Location, where $X_{i}$ and $Y_{i}$ are the UAV coordinates
$L(X_{r},Y_{r})$	Location for the battery replacement, where $X_{r}$ and $Y_{r}$ are the batery replacement coordinates
$R_{max}$	Radio Range
$route_{RSN,DN}$	route between source and destination nodes via2 multiple relay nodes
$RE_{i}$	Remaining energy of each UAV
$RN_{i}$	Relay node for a path between the source SN and destination DN
$s_{i}$	UAV speed
$S_{max}$	Maximum speed limit
SN	Source node
$t_{move}$	Time needed to fly from the current location to the event location
$traj_{i}$	UAV mobility trajectory, which can be Stay-At, Way-point, Eight, Scan, and Oval movements
$v_{i}$	A given UAV with an individual node identity *i*, where 1 < *i* < *n*
